# Incidence of pregnancy-associated acute kidney injury in low- and middle-income countries: a meta-analysis

**DOI:** 10.2471/BLT.24.293077

**Published:** 2025-06-24

**Authors:** Phu Nguyen Trong Tran, Anyarin Wannakittirat, Valerie Luyckx, Kate Wiles, Manjusha Yadla, Rajasekara Chakravarthi, Marlies Ostermann, Vin-Cent Wu, Ravindra L Mehta, Nattachai Srisawat

**Affiliations:** a Center of Excellence in Critical Care Nephrology and Division of Nephrology, Faculty of Medicine, Chulalongkorn University, Bangkok 10400, Thailand.; b Division of Nephrology, Faculty of Medicine, Naresuan University Hospital, Phitsanulok, Thailand.; c University Children’s Hospital, University of Zurich, Zurich, Switzerland.; d Department of Maternal Medicine, Queen Mary University of London, London, England.; e Department of Nephrology, Gandhi Medical College, Hyderabad, India.; f Nephrology and Transplant Services, Yashoda Hospitals, Hyderabad, India.; g Department of Critical Care and Nephrology, King’s College London, London, England.; h Division of Nephrology, Department of Internal Medicine, National Taiwan University Hospital, Taipei, Taiwan.; i Division of Nephrology, University of California San Diego, San Diego, United States of America.

## Abstract

**Objective:**

To conduct a systematic review and meta-analysis of pregnancy-associated acute kidney injury in low- and middle-income countries.

**Method:**

We searched the databases Cochrane Central Register of Controlled Trials, Embase, Google Scholar, OvidMedline, ProQuest and Scopus for articles published during 2013–2025 reporting the incidence, etiology and outcomes of the condition in low- and middle-income countries. We conducted a meta-analysis of the studies that used the diagnostic criteria of the Kidney Disease: Improving Global Outcomes organization. We conducted subgroup analyses and a meta-regression to explore sources of heterogeneity.

**Findings:**

We reviewed 43 studies and included 40 in our meta-analysis, covering 424 081 pregnancies in 15 low- and middle-income countries. We observed a pooled incidence of 91 cases (95% confidence interval, CI: 63–133) per 10 000 pregnancies, highest in studies conducted in the World Health Organization African Region (254; 95% CI: 152–421). We estimated case fataliy of 10.8% (95% CI: 7.6–15.3) and neonatal death or stillbirth in 29.8% of cases (95% CI: 24.2–36.1). We observed that the condition was associated with 18.8-fold higher odds of maternal death (95% CI: 10.0–35.5) and 4.6-fold higher odds of adverse fetal outcomes (95% CI: 2.1–10.0). We identified pre-eclampsia (44.1%), haemorrhage (26.2%) and sepsis (16.5%) as the leading etiologies.

**Conclusion:**

Pregnancy-associated acute kidney injury is a significant maternal health concern in low- and middle-income countries. By providing more resources to prevent the common etiologies and expand the availability of antenatal care, its deleterious effects on maternal and fetal outcomes can be reduced.

## Résumé

**Objectif:**

Réaliser une revue systématique et une méta-analyse des lésions rénales aiguës associées à la grossesse dans les pays à revenu faible et intermédiaire.

**Méthode:**

Nous avons recherché dans les bases de données Central Cochrane, Embase, Google Scholar, OvidMedline, ProQuest et Scopus des articles publiés entre 2013 et 2025 faisant état de l’incidence, de l’étiologie et des résultats de cette affection dans les pays à revenu faible ou intermédiaire. Nous avons réalisé une méta-analyse des études qui appliquaient les critères de diagnostic de l’organisation Kidney Disease: Improving Global Outcomes. Nous avons effectué des analyses de sous-groupes et une métarégression afin d’explorer les sources d’hétérogénéité.

**Résultats:**

Nous avons examiné 43 études et en avons inclus 40 dans notre méta-analyse, couvrant 424 081 grossesses dans 15 pays à revenu faible ou intermédiaire. Nous avons observé une incidence groupée de 91 cas (intervalle de confiance (IC) à 95%: 63–133) pour 10 000 grossesses, la plus élevée étant observée dans les études menées dans la région Afrique de l’Organisation mondiale de la Santé (254; IC à 95%: 152–421). Nous avons estimé la mortalité maternelle à 10,8% (IC à 95%: 7,6–15,3) et le décès néonatal ou la mortinaissance à 29,8% des cas (IC à 95%: 24,2–36,1). Nous avons observé que les lésions rénales aiguës étaient associées à un risque 18,8 fois plus élevé de décès maternel (IC à 95%: 10,0–35,5) et à un risque 4,6 fois supérieur d’issues défavorables pour le fœtus (IC à 95%: 2,1–10,0). Nous avons identifié la pré-éclampsie (44,1%), l’hémorragie (26,2%) et la septicémie (16,5%) parmi les principales étiologies.

**Conclusion:**

Les lésions rénales aiguës associées à la grossesse constituent un problème de santé maternelle important dans les pays à revenu faible ou intermédiaire. En fournissant davantage de ressources pour prévenir les étiologies courantes et en élargissant l’accès aux soins prénatals, il est possible de réduire le fardeau et les effets sur les résultats pour la mère et le fœtus.

## Resumen

**Objetivo:**

Realizar una revisión sistemática y un metanálisis sobre la lesión renal aguda asociada al embarazo en países de ingresos bajos y medios.

**Método:**

Se efectuó una búsqueda en las bases de datos Central Cochrane, Embase, Google Scholar, OvidMedline, ProQuest y Scopus de artículos publicados entre 2013 y 2025 que informaran sobre la incidencia, etiología y desenlaces de esta afección en países de ingresos bajos y medianos. Se llevó a cabo un metanálisis de los estudios que utilizaron los criterios diagnósticos de la organización Kidney Disease: Improving Global Outcomes. Se realizaron análisis por subgrupos y una metarregresión para explorar fuentes de heterogeneidad.

**Resultados:**

Se revisaron 43 estudios, de los cuales 40 fueron incluidos en el metanálisis, abarcando un total de 424 081 embarazos en 15 países de ingresos bajos y medios. Se observó una incidencia agrupada de 91 casos (intervalo de confianza, IC del 95%: 63-133) por cada 10 000 embarazos, con la cifra más alta en los estudios realizados en la Región de África de la Organización Mundial de la Salud (254; IC del 95%: 152-421). Se estimó una mortalidad materna del 10,8% (IC del 95%: 7,6-15,3) y una tasa de muerte neonatal o mortinatalidad en el 29,8% de los casos (IC del 95%: 24,2-36,1). Se identificó una asociación de esta afección con una probabilidad 18,8 veces mayor de muerte materna (IC del 95%: 10,0-35,5) y 4,6 veces mayor de desenlaces fetales adversos (IC del 95%: 2,1-10,0). Las principales etiologías fueron la preeclampsia (44,1%), las hemorragias (26,2%) y la sepsis (16,5%).

**Conclusión:**

La lesión renal aguda asociada al embarazo representa un problema relevante de salud materna en países de ingresos bajos y medios. La asignación de mayores recursos para prevenir las etiologías más frecuentes y ampliar la cobertura de la atención prenatal permitiría reducir su carga y sus efectos negativos sobre los desenlaces maternos y fetales.

## ملخص

الغرض إجراء مراجعة منهجية وتحليل تلوي لإصابة الكلى الحادة المرتبطة بالحمل في الدول ذات الدخل المنخفض والدخل المتوسط.

الطريقة قمنا بالبحث في قواعد بيانات Central Cochrane، وEmbase، وGoogle Scholar، وOvidMedline، وProQuest، وScopus، عن مقالات نُشرت خلال الفترة 2013 إلى 2025، تتناول معدل الإصابة، وأسبابها، ونتائجها في الدول منخفضة ومتوسطة الدخل. وقمت بإجراء تحليل تلوي للدراسات التي استخدمت المعايير التشخيصية لمؤسسة "أمراض الكلى: مؤسسة تحسين النتائج العالمية". كما أجرينا تحليلات للمجموعات الفرعية، وتحوفًا تلويًا لاستكشاف مصادر التباين.

النتائج قمنا بمراجعة 43 دراسة، وأدرجنا 40 منها في تحليلنا التلوي، الذي غطى 424081 من حالات الحمل في 15 دولة منخفضة ومتوسطة الدخل. لاحظنا معدلًا مُجمّعًا بلغ 91 حالة (بفاصل ثقة %95: 63 إلى 133) لكل 10000 حالة حمل، وهو أعلى معدل في الدراسات التي أُجريت في منطقة أفريقيا التابعة لمنظمة الصحة العالمية (254 حالة؛ بفاصل ثقة %95: 152 إلى 421). وقدّرنا معدل وفيات الأمهات بنسبة %10.8 (بفاصل ثقة %95: 7.6 إلى 15.3) ووفاة حديثي الولادة أو ولادة جنين ميت في %29.8 من الحالات (بفاصل ثقة %95: 24.2 إلى 36.1). ولاحظنا أن هذه الحالة ارتبطت بزيادة احتمالات وفاة الأم بمقدار 18.8 ضعفًا (بفاصل ثقة %95: 10.0 إلى 5.5) وزيادة احتمالات حدوث نتائج سلبية على الجنين بمقدار 4.6 ضعفًا (بفاصل ثقة %95: 2.1 إلى 10.0). حددنا مقدمات الارتعاج (%44.1)، والنزيف (%26.2)، وتسمم الدم (%16.5) كأسباب رئيسية.

النتائج تُعدّ إصابة الكلى الحادة المرتبطة بالحمل مصدر قلق كبير لصحة الأم في الدول ذات الدخل المنخفض والمتوسط. ومن خلال توفير المزيد من الموارد للوقاية من الأسباب الشائعة وتوسيع نطاق رعاية ما قبل الولادة، يُمكن تخفيف العبء والآثار على نتائج الأم والجنين.

## 摘要

**目的:**

旨在对中低收入国家的妊娠相关急性肾损伤进行系统评价和荟萃分析。

**方法:**

我们搜索了 Central Cochrane、Embase、Google Scholar、OvidMedline、ProQuest 和 Scopus 这六个数据库，以查找 2013 年至 2025 年期间发表的报告了中低收入国家妊娠相关急性肾损伤发病率、病因和结局的文章。我们对采用改善全球肾脏病预后组织诊断标准的相关研究进行了荟萃分析。我们通过亚组分析和荟萃回归探索了异质性来源。

**结果:**

我们回顾了共 43 项研究，并将其中 40 项研究（涵盖了 15 个中低收入国家中的 424,081 名孕妇）纳入了荟萃分析。我们发现，每 10,000 名孕妇中的合并发病率为 91 例【95% 置信区间 (CI)：63-133】，其中在世界卫生组织非洲区域所开展研究中报告的发病率最高（254 例；95% CI：152-421）。据我们估计，在所有病例中，孕产妇死亡率为 10.8%（95% CI：7.6-15.3），新生儿死亡率或死产率为 29.8%（95% CI：24.2-36.1）。我们发现，妊娠相关急性肾损伤导致孕产妇死亡几率提高了 18.8 倍（95% CI：10.0-35.5），不良胎儿结局几率提高了 4.6 倍（95% CI：2.1-10.0）。我们发现，主要的病因包括先兆子痫（占 44.1%）、产后出血（占 26.2%）和脓毒症（占 16.5%）。

**结论:**

妊娠相关急性肾损伤是中低收入国家普遍存在的一个重大的孕产妇健康问题。中低收入国家可通过投入更多资源以避免常见病因和提高产检服务的可及性，从而减轻因孕产妇和胎儿出现不良结局而对该国所造成的负担并降低这些不良结局的影响。

## Резюме

**Цель:**

Выполнить систематический обзор и метаанализ случаев гестационного острого поражения почек в странах с низким и средним уровнем дохода.

**Методы:**

Авторы провели поиск в базах данных Central Cochrane, Embase, Google Scholar, OvidMedline, ProQuest и Scopus на предмет статей, опубликованных в период с 2013 по 2025 год и содержащих данные о случаях, этиологии и исходах этого заболевания в странах с низким и средним уровнем дохода. Был проведен метаанализ исследований, в которых использовались диагностические критерии Международной организации по улучшению исходов заболеваний почек (Kidney Disease: Improving Global Outcomes). Был проведен анализ по подгруппам, а также была просчитана метарегрессия для выявления источников гетерогенности данных.

**Результаты:**

Из 43 рассмотренных исследований в метаанализ были включены 40, что позволило обобщить данные о 424 081 случае беременности в 15 странах с низким и средним уровнем дохода. Совокупная заболеваемость составила 91 случай (95%-й ДИ: 63–133) на 10 000 беременностей с наибольшими значениями в тех исследованиях, которые проводились в Африканском регионе Всемирной организации здравоохранения (254; 95%-й ДИ: 152–421). Оценка материнской смертности была на уровне 10,8% (95%-й ДИ: 7,6–15,3), а смерть новорожденных или мертворожденность наблюдалась в 29,8% случаев (95%-й ДИ: 24,2–36,1). По наблюдениям авторов, изучаемое заболевание ассоциировалось с ростом вероятности смерти матери в 18,8 раза (95%-й ДИ: 10,0–35,5) и неблагоприятного исхода для плода в 4,6 раза (95%-й ДИ: 2,1–10,0). Авторы выяснили, что основными этиологиями были: преэклампсия (44,1%), кровотечение (26,2%) и сепсис (16,5%).

**Вывод:**

Гестационное острое поражение почек представляет серьезную угрозу материнскому здоровью в странах с низким и средним уровнем дохода. Выделение большего количества ресурсов на предотвращение распространенных этиологий данного заболевания и расширение доступности дородового ухода позволит снизить бремя и уменьшить влияние на исходы для матери и плода.

## Introduction

Pregnancy-associated acute kidney injury is defined as a sudden decline in kidney function during pregnancy or the postpartum period (6–8 weeks after giving birth). Accounting for 1% of all acute kidney injury admissions globally in women of reproductive age, dialysis administered for severe acute kidney injury is most often for the pregnancy-associated type.^1^ The consequences of the condition are severe, with case fatality rates ranging from 9.3% (29/313) in Tunisia to 28.3% (13/46) in Morocco, and increased risks of preterm birth, stillbirth and neonatal intensive care admissions.^2^ Given its significant impact, pregnancy-associated acute kidney injury is widely recognized as an urgent public health issue, as a major contributor to maternal and fetal morbidity and mortality.^3^

The burden of pregnancy-associated acute kidney injury is higher in low- and middle-income countries compared to high-income countries. Large registry-based studies conducted in Canada (2003–2010)^4^ and the United States of America (1999–2011)^5^ have reported an incidence of approximately 2–8 cases per 10 000 pregnancies. In contrast, studies in Benin, China, India, Morocco and South Africa have shown incidences from 7 to over 1000 cases per 10 000 pregnancies.^6^^–^^12^ Although case fatality rates in women with the condition is estimated to be around 3% in high-income countries, this figure has exceeded 20% in India, Nigeria and the United Republic of Tanzania.^5^^,^^13^^–^^15^ This disparity is further reflected in the distribution of common etiologies. For example, infection-related severe maternal outcomes are reported to be more than 15 times more frequent in low- and middle-income countries than in high-income countries,^16^ and maternal deaths as a result of pre-eclampsia are significantly more prevalent in socioeconomically disadvantaged populations of low- and middle-income countries.^17^ These findings highlight the need for focused research and public health interventions to address pregnancy-related acute kidney injury in low- and middle-income countries.

Despite increasing awareness of the health burden, research in low- and middle-income countries remains limited; in contrast to high-income countries, most do not have a national registry for the condition, and most studies have been small-scale cohort studies or case reports.^18^ The true burden of the condition in lower-income countries is difficult to determine because of inconsistent study design, variable diagnostic criteria and heterogeneous reporting. Although a previous review estimated global incidence, the burden and etiology specifically for low- and middle-income countries were unavailable.^19^ One review highlighted clinical profiles and progress in low- and middle-income countries, but lacked a systematic evaluation of the evidence.^1^ More importantly, no previous work has attempted to address interstudy variability and ensure generalizability by comprehensively assessing incidence and outcomes in low- and middle-income countries using harmonized diagnostic criteria.

In this study we seek to address these knowledge gaps by conducting a comprehensive meta-analysis of pregnancy-associated acute kidney injury in low- and middle-income countries. Using criteria defined by the Kidney Disease: Improving Global Outcomes organization to standardize case definitions across diverse populations, we aim to characterize the burden of the condition by assessing its incidence and impact on maternal and fetal outcomes, as well as identify potential opportunities for intervention by analysing etiologies and modifiable health-care factors. 

## Methods

### Outcome of interest

The primary outcome of interest from our meta-analysis is the burden of pregnancy-associated acute kidney injury, assessed by its incidence and associated maternal and fetal outcomes. We define incidence as the total number of new cases observed per 10 000 pregnancies during the study period. Maternal outcomes include renal replacement therapy, chronic or end-stage kidney disease and death. Fetal outcomes include neonatal death and stillbirth.

Secondary outcomes include etiologies, as defined by the original studies. Sepsis encompasses post-abortal and puerperal infections; pre-eclampsia spectrum includes gestational hypertension, pre-eclampsia, severe pre-eclampsia and eclampsia, as well as haemolysis, elevated liver enzymes and low platelet count (HELLP syndrome); and obstetric haemorrhage includes both antepartum, intrapartum and postpartum bleeding.

### Search

We registered our systematic review and meta-analysis protocol on PROSPERO (CRD42024512357). We conducted a comprehensive search for both peer-reviewed publications and unpublished studies in the databases Cochrane Central Register of Controlled Trials, Embase, Google Scholar, OvidMedline, ProQuest and Scopus, published or released during the period 1 January 2013 to 15 March 2025, on 16 March 2025. Our search strategy was based on the two primary concepts of acute kidney injury and pregnancy, incorporating various keyword variations (details available in online repository).^20^

We screened observational studies conducted in low- and middle-income countries (as defined by the World Bank)^21^ during 2013–2023 that reported the primary outcome of the incidence of pregnancy-associated acute kidney injury in any language. To ensure generalizability, we required that studies selected for review included both pregnancy and the postpartum period. We excluded studies that focused solely on subgroups (e.g. sepsis or pre-eclampsia), lacked diagnostic criteria for pregnancy-associated acute kidney injury or defined the condition solely by the need for dialysis. Summaries of the excluded studies are provided in the online repository.^20^

We uploaded all resulting citations to Covidence (Veritas Health Innovation Ltd, Melbourne, Australia), where duplicates were automatically removed. Two authors independently screened titles and abstracts, with a third author resolving disagreements. We assessed full texts according to our inclusion and exclusion criteria. We extracted data including study details (authors, year, duration, country, design, setting), demographics (age, pregnancy duration), sample size, diagnostic criteria, etiology and outcomes. Two authors performed data extraction independently, and a third author verified the extracted data.

### Exploration of heterogeneity

To explore factors contributing to heterogeneity in the pooled analysis, we supplemented study-level characteristics (e.g. design and quality) with country-level socioeconomic and geographical metadata. Geographically, we grouped studies by country and by World Health Organization (WHO) regions.^22^ We defined socioeconomic context using the World Bank income classification at the time of each study.^21^ We also categorized studies by national health-care expenditure as a percentage of gross domestic product (GDP) – either less than 5% or 5–10% – based on WHO statistics.^23^^,^^24^ For antenatal care coverage, we used United Nations Children’s Fund data on the percentage of women aged 15–49 years receiving at least one or at least four antenatal visits,^25^ categorized as less than 75%, or 75% or more.

We used the Joanna Briggs Institute critical appraisal tools for studies reporting prevalence or incidence data to assess the risk of bias and study quality.^26^ According to a score ranging from 0 to 9, we classified study quality as low (0–3), medium (4–6) and high (7–9; online repository).^20^

### Data analysis

We conducted a meta-analysis of proportions using the metaprop function of the meta and metafor packages of RStudio version 4.3.1 (Posit PBC, Boston, USA), which applies a random-intercept logistic regression (generalized linear mixed model) to logit-transformed proportions. We calculated pooled odds ratios, 95% confidence intervals (CIs) and *P*-values for binary outcomes (maternal or fetal mortality) by the inverse variance method and Wald z-test of the metabin function of RStudio. We based all analyses on random-effect models to account for between-study variability.

We assessed heterogeneity using the *I*^2^ statistic, with values of 70% and higher indicating substantial heterogeneity. To explore potential sources, we conducted subgroup analyses (for study- and country-level categorical factors) and meta-regression (for the middle year of data collection as a continuous moderator) using the *Q*-test (*χ^2^* test) and Wald *z*-test, respectively. We performed an interaction term analysis between the middle year and country socioeconomic factors using a multivariable meta-regression of the metareg function in RStudio, adjusted for study design and quality.

Our sensitivity analyses included leave-one-out influence analysis, and a stepwise exclusion of low- and medium-quality studies and those with sample sizes smaller than 5000 and 10 000. We compared the pooled estimates and *I*^2^ values to assess robustness.

We could not disaggregate data by age because original data were unavailable. 

## Results

### Study population

**Fig. 1:**
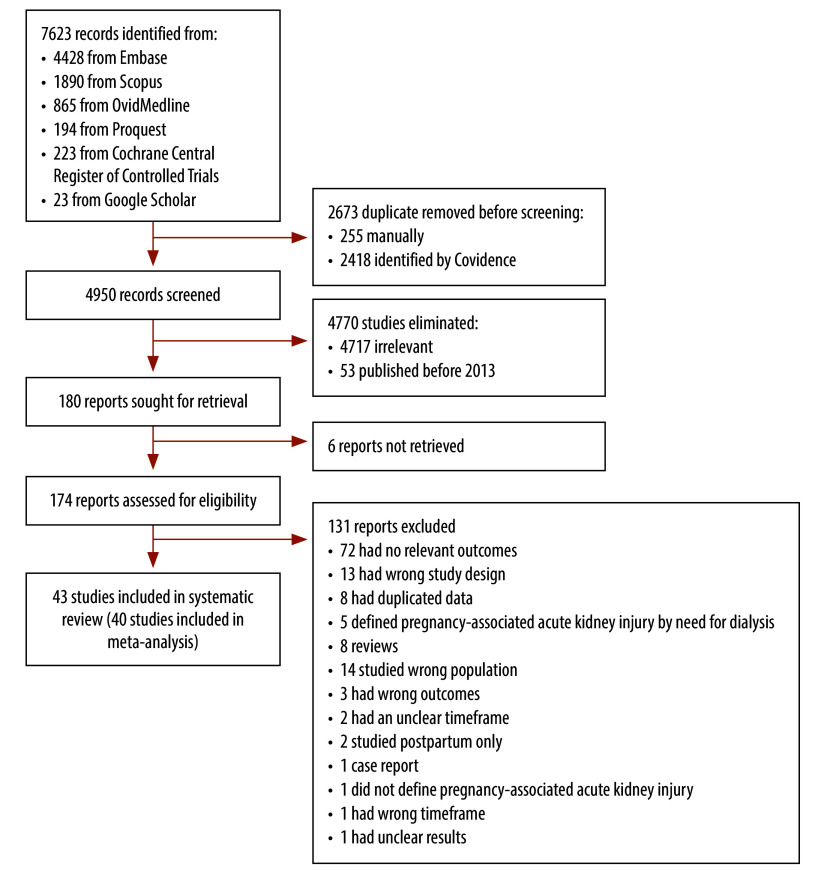
Flowchart of the selection of studies on pregnancy-associated acute kidney injury in low- and middle-income countries

After screening 4950 records, we assessed 174 articles for eligibility; on excluding 131 articles, we reviewed 43 studies (Fig. 1). Our included studies were published from 2015 to 2024 and reported the incidence of pregnancy-associated acute kidney injury from 2013 to 2023. Most studies were conducted in India (19);^6^^,^^7^^,^^15^^,^^27^^–^^42^ five were from China^8^^,^^43^^–^^46^ and three from Egypt.^47^^–^^49^ Two studies were conducted in each of Nepal,^50^^,^^51^ Nigeria^13^^,^^52^ and the United Republic of Tanzania;^14^^,^^53^ and a single study was conducted in each of Bangladesh,^54^ Benin,^12^ Chad,^55^ Ethiopia,^56^ Guinea,^57^ Kenya,^58^ Malawi,^59^ the Philippines,^60^ Romania^61^ and Somalia^62^ (Table 1).

**Table 1:** Characteristics of the studies included in a systematic review and meta-analysis of pregnancy-associated acute kidney injury in low- and middle-income countries, published during 2015–2024

Study	Country	Study period, study design^a^	Study period (months)	Sample size (no. pregnancies)	No. of cases^b^	Incidence per 10 000 pregnancies	Study quality^c^
Bhargava et al., 201^61^	India	2013–2014	12	371	21	566	7
Paudyal et al., 2015^51^	Nepal	2013–2014	18	7 108	15	21	6
Hounkponou et al., 2017^12^	Benin	2016–2016	6	324	38	1173	6
Huang & Chen, 2017^43^	China	2013–2017^ d^	90	42 173	343^ e^	81	7
Chitale et al., 2018^30^	India	2014–2017	48	1 057	26	246	4
Cooke et al., 2018^59^	Malawi	2015–2015	4	2 300	26	113	8
Gao et al., 2018^44^	China	2014–2017^ d^	42	15 637	82	52	7
Prakash et al., 2018^31^	India	2014–2016	20	4 741	132	278	7
Sharma et al., 2018^15^	India	2017–2017	6	1 600	22	138	5
Ganesh & Soujanya, 2019^32^	India	2016–2018	28	18 000	36	20	5
Kivai et al., 2019^58^	Kenya	2018–2018	2	2 068	66	319	7
Liu et al., 2019^8^	China	2013–2015^ d^	36	10 920	720	659	9
Thakur et al., 2019^50^	Nepal	2015–2015	12	11 490	28	24	6
Bah et al., 2020^57^	Guinea	2018–2019	6	2 438	56	230	3
Elshinnawy et al., 2020^47^	Egypt	2017–2017	12	13 050	78	60	6
Gayathiri et al., 2020^33^	India	2018–2019	12	9 921	18	18	6
Papegowda et al., 2020^7^	India	2014–2019^ d^	60	50 735	36	7	3
Saini et al., 2020^34^	India	2015–2016	24	5 645	81	143	3
Gaber et al., 2021^48^	Egypt	2017–2019	24	4 500	40	89	3
Kanmani et al., 2021^35^	India	2020–2020	12	9 099	21	23	4
Li et al., 2021^45^	China	2015–2018^ d^	48	6 512	136	209	7
Meca et al., 2021^61^	Romania	2020–2021^ d^	20	4 000	18	45	6
Li et al., 2022^46^	China	2013–2020^ d^	84	71 763	157	22	7
Mandal et al., 2022^37^	India	2021–2021	12	9 270	79	85	7
Praba & Gomathi, 2022^38^	India	2020–2021^ d^	24	9 444	34	36	6
Ruggajo et al., 2022^53^	United Republic of Tanzania	2015–2016	6	5 448	99	182	9
Sachan et al., 2022^39^	India	2019–2020	16	14 702	150	102	7
Abderraman et al., 2023^55^	Chad	2020–2020	6	1 238	56	452	5
Anitha et al., 2023^40^	India	2020–2020	12	7 409	104	140	5
Anvar et al., 2023^27^	India	2015–2020^ d^	52	33 403	70	21	7
Faisal et al., 2023^54^	Bangladesh	2018–2019	12	351	32^ e^	912	5
Kharkongor et al., 2023^41^	India	2022–2022	12	10 138	110	109	7
Rage et al., 2023^62^	Somalia	2020–2022^ d^	24	9 457	31	33	6
Rajanna et al., 2023^42^	India	2018–2019	12	2 650	42	158	5
Thakur et al., 2023^36^	India	2018–2019	12	6 024	47	78	6
Berhe et al., 2024^56^	Ethiopia	2017–2021^ d^	60	27 350	176	64	7
Habib et al., 2024^13^	Nigeria	2021–2023^ d^	24	1 647	103	625	7
Ismael et al., 2024^49^	Egypt	2022–2023^ d^	18	4 390	18	41	6
Kamal, 2024^28^	India	2022–2023^ d^	12	21 234	172^ e^	81	7
Mahalingam et al., 2024^29^	India	2019–2019^ d^	12	16 469	19	12	6
Reston & Ti, 2024^60^	Philippines	2019–2021^ d^	36	3 556	46	129	6
Shija et al., 2024^14^	United Republic of Tanzania	2019–2023	48	4 007	51	127	6
Waziri et al., 2024^52^	Nigeria	2019–2022	34	4 200	113	269	8

^a^ Study design prospective, unless otherwise indicated.

^b^ Pregnancy-associated acute kidney injury diagnosed according to the definitions included in the online repository.^20^ Unless otherwise indicated, all studies used criteria defined by: Kidney Disease: Improving Global Outcomes; Acute Kidney Injury Network (AKIN); and/or Risk, Injury, Failure, Loss of kidney function, End-stage kidney disease (justifying both acute elevation of serum creatinine and urine output).

^c^ We used the Joanna Briggs Institute critical appraisal tools for studies reporting prevalence or incidence data to assess the risk of bias and study quality.^26^ We deemed a score of 0–3 as low quality, 4–6 as medium and 7–9 as high.

^d^ Study design retrospective.

^e^ Pregnancy-associated acute kidney injury diagnosed according to serum creatinine only (i.e. not included in meta-analysis).

We observed that diagnostic criteria for pregnancy-associated acute kidney injury included those of the Kidney Disease: Improving Global Outcomes organization in 40 studies^6^^–^^8^^,^^12^^–^^15^^,^^27^^,^^29^^–^^42^^,^^44^^–^^53^^,^^55^^–^^62^ and serum creatinine concentration in pregnancy in three studies.^28^^,^^43^^,^^54^ We summarize details regarding diagnostic criteria conversion in the online repository.^20^ In assigning study quality, the most common reasons for point deduction were insufficient information and data analysis for patients without pregnancy-associated acute kidney injury (Table 1). To ensure comparability between studies, we only included the 40 studies using the diagnostic criteria of the Kidney Disease: Improving Global Outcomes organization in our meta-analysis.

### Incidence

**Fig. 2:**
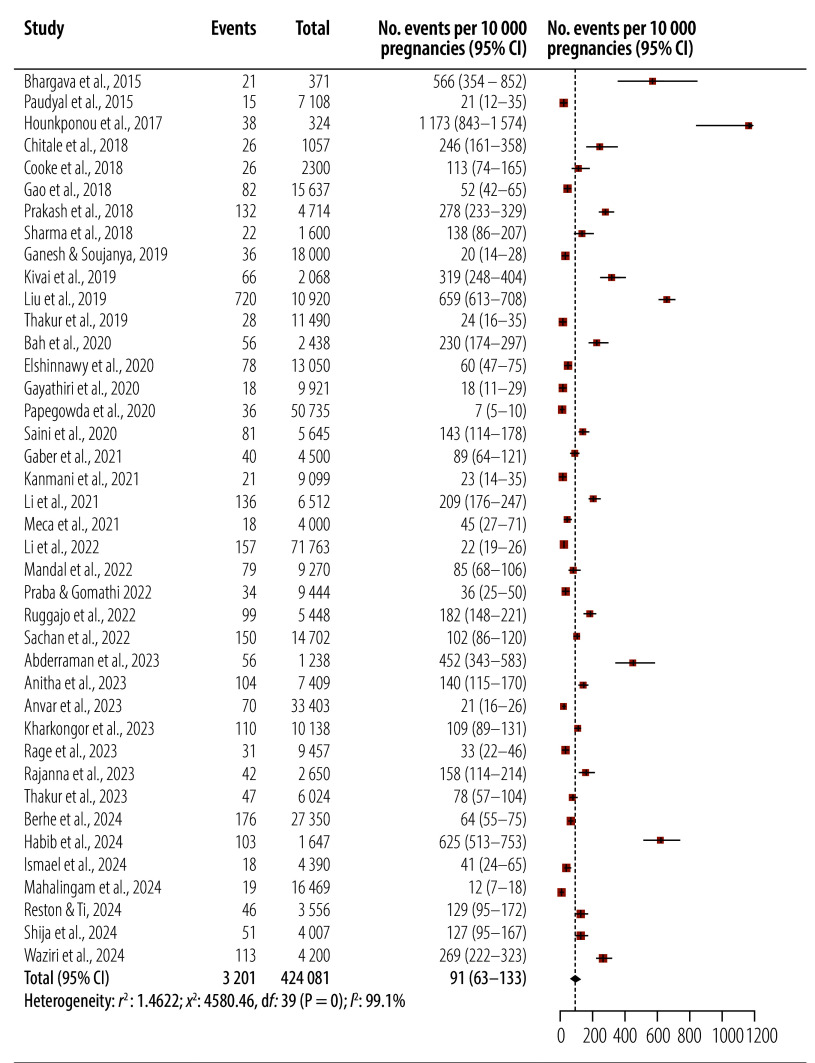
Study events and pooled incidence of pregnancy-associated acute kidney injury in low- and middle-income countries

We analysed a total of 3201 cases of pregnancy-induced acute kidney injury from 424 081 pregnancies across the 40 studies included in the meta-analysis. We observed a pooled incidence of 91 cases per 10 000 pregnancies (95% CI: 63–133; *I*^2^: 99.1%; Fig. 2), ranging from 7^7^ in India to 1173^12^ in Benin per 10 000 pregnancies (Table 1).

CI: confidence interval.

We noted that prospective studies (118 cases per 10 000 pregnancies; 95% CI: 79–177; *I*^2^: 97.6%)^6^^,^^12^^–^^15^^,^^30^^–^^37^^,^^39^^–^^42^^,^^47^^,^^48^^,^^50^^–^^53^^,^^55^^,^^57^^–^^59^ had higher estimates than retrospective studies (56; 95% CI: 28–112; *I*^2^: 99.6%),^7^^,^^8^^,^^13^^,^^27^^,^^29^^,^^38^^,^^44^^–^^46^^,^^49^^,^^56^^,^^60^^–^^62^ although studies recruiting only intensive care unit patients reported lower estimates (44; 95% CI: 3–532; *I*^2^: 99.7%)^7^^,^^52^ than studies recruiting patients from all wards (95; 95% CI: 66–137; *I*^2^: 99.1%).^6^^,^^8^^,^^12^^–^^15^^,^^27^^,^^29^^–^^42^^,^^44^^–^^51^^,^^53^^,^^55^^–^^62^

When studies are pooled by country, we observed the lowest incidence per 10 000 pregnancies in Nepal (23; 95% CI: 17–31),^50^^,^^51^ Somalia (33; 95% CI: 22–46)^62^ and Romania (45; 95% CI: 27–71);^61^ and the highest in Benin (1173; 95% CI: 843–1574),^12^ Chad (452; 95% CI: 343–483)^55^ and Nigeria (409; 95% CI: 227–729; *I*^2^: 97.5%; Table 2).^13^^,^^52^ Among the WHO regions, we observed the highest incidence in pooled studies based in the African Region (254; 95% CI: 152–421; *I*^2^: 98.4%)^12^^–^^14^^,^^52^^,^^53^^,^^55^^–^^59^ and the Western Pacific Region (116; 95% CI: 41–323; *I*^2^: 99.8%).^8^^,^^44^^–^^46^^,^^60^ We noted that pooled incidence per 10 000 pregnancies in the South-East Asia and Eastern Mediterranean regions were similar at 59 (95% CI: 36–99; *I*^2^: 98.2%)^6^^,^^7^^,^^15^^,^^27^^,^^29^^–^^42^^,^^50^^,^^51^ and 52 (95% CI: 36–76; *I*^2^: 84.7%),^47^^–^^49^^,^^62^ respectively (Table 2).

**Table 2:** Heterogeneity in prevalence of pregnancy-associated acute kidney injury in low- and middle-income countries

Characteristic (*P*-value)	No. studies in meta-analysis	No. cases	No. pregnancies	Pooled incidence per 10 000 pregnancies (95% CI)	*I*^2^, %
**Overall**	40^6^^–^^8^^,^^12^^–^^15^^,^^27^^,^^29^^–^^42^^,^^44^^–^^53^^,^^55^^–^^62^	3 201	424 081	91 (63–133)	99.1
**Study design (0.0689)**					
Prospective	26^6^^,^^12^^–^^15^^,^^30^^–^^37^^,^^39^^–^^42^^,^^47^^,^^48^^,^^50^^–^^53^^,^^55^^,^^57^^–^^59^	1 555	158 798	118 (79–177)	97.6
Retrospective	14^7^^,^^8^^,^^13^^,^^27^^,^^29^^,^^38^^,^^44^^–^^46^^,^^49^^,^^56^^,^^60^^–^^62^	1 646	424 081	56 (28–112)	99.6
**Recruiting site (0.5544)**					
Intensive care unit	2^7^^,^^52^	149	54 935	44 (3–532)	99.7
All wards	38^6^^,^^8^^,^^12^^–^^15^^,^^27^^,^^29^^–^^42^^,^^44^^–^^51^^,^^53^^,^^55^^–^^62^	3 052	369 146	95 (66–137)	99.1
**WHO region (< 0.001)**					
African	10^12^^–^^14^^,^^52^^,^^53^^,^^55^^–^^59^	784	51 020	254 (152–421)	98.4
South-East Asia	20^6^^,^^7^^,^^15^^,^^27^^,^^29^^–^^42^^,^^50^^,^^51^	1 091	229 276	59 (36–99)	98.2
European	1^61^	18	4 000	45 (27–71)	–
Eastern Mediterranean	4^47^^–^^49^^,^^62^	167	31 397	52 (36–76)	84.7
Western Pacific	5^8^^,^^44^^–^^46^^,^^60^	1 141	108 388	116 (41–323)	99.8
**Country (< 0.001)**					
Benin	1^12^	38	324	1 173 (843–1 574)	–
Chad	1^55^	56	1 238	452 (343–583)	–
China	4^8^^,^^44^^–^^46^	1 095	104 832	113 (31–405)	99.8
Egypt	3^47^^–^^49^	136	21 940	61 (45–84)	75.9
Ethiopia	1^56^	176	27 350	64 (55–75)	–
Guinea	1^57^	56	2 438	230 (174–297)	–
India	18^6^^,^^7^^,^^15^^,^^27^^,^^29^^–^^42^	1 048	210 678	66 (38–114)	98.3
Kenya	1^58^	66	2 068	319 (248–404)	–
Malawi	1^59^	26	2 300	113 (74–165)	–
Nepal	2^50^^,^^51^	43	18 598	23 (17–31)	0.0
Nigeria	2^13^^,^^52^	216	5 847	409 (227–729)	97.5
Philippines	1^60^	46	3 556	129 (95–172)	–
Romania	1^61^	18	4 000	45 (27–71)	–
Somalia	1^62^	31	9 457	33 (22–46)	–
United Republic of Tanzania	2^14^^,^^53^	150	9 455	155 (121–198)	76.9
**Income level (0.8404)**					
Upper-middle	5^8^^,^^44^^–^^46^^,^^61^	1 113	108 832	94 (32–278)	99.8
Lower-middle	26^6^^,^^7^^,^^13^^–^^15^^,^^27^^,^^29^^–^^42^^,^^47^^–^^49^^,^^52^^,^^58^^,^^60^	1 563	248 096	84 (54–131)	98.4
Low	9^12^^,^^50^^,^^51^^,^^53^^,^^55^^–^^57^^,^^59^^,^^62^	525	67 153	113 (48–264)	98.6
**Percentage of GDP spent on health care (0.7249)**					
5–10	9^45^^–^^48^^,^^50^^,^^55^^,^^59^^–^^61^	585	118 409	82 (44–152)	98.7
< 5	31^6^^–^^8^^,^^12^^–^^15^^,^^27^^,^^29^^–^^42^^,^^44^^,^^49^^,^^51^^–^^53^^,^^56^^–^^58^^,^^62^	2 616	305 672	94 (60–147)	99.2
**Percentage of women receiving at least one antenatal care visit (0.2855)**					
≥ 75	32^6^^–^^8^^,^^14^^,^^15^^,^^27^^,^^29^^–^^42^^,^^44^^–^^50^^,^^53^^,^^57^^–^^60^	2 651	368 757	81 (55–119)	99.2
< 75	8^12^^,^^13^^,^^51^^,^^52^^,^^55^^,^^56^^,^^61^^,^^62^	550	55 324	146 (54–390)	99.1
**Percentage of women receiving at least four antenatal care visits (0.4371)**					
≥ 75	8^8^^,^^12^^,^^44^^–^^49^^,^^60^	1 261	130 328	91 (45–183)	99.7
< 75	32^6^^,^^7^^,^^13^^–^^15^^,^^27^^,^^29^^–^^42^^,^^50^^–^^53^^,^^55^^–^^59^^,^^61^^,^^62^	1 924	293 753	91 (59–141)	98.5
**Sensitivity analysis: study quality**					
Medium- and high-quality only	36^6^^,^^8^^,^^12^^–^^15^^,^^27^^,^^29^^–^^33^^,^^35^^–^^42^^,^^44^^–^^53^^,^^55^^,^^56^^,^^58^^–^^62^	2 988	360 763	94 (64–139)	99.2
High-quality only	16^6^^,^^8^^,^^13^^,^^27^^,^^31^^,^^37^^,^^39^^,^^41^^,^^44^^–^^46^^,^^52^^,^^53^^,^^56^^,^^58^^,^^59^	2 240	220 470	144 (86–241)	99.5
**Sensitivity analysis: sample size**					
≥ 5 000 pregnancies	23^7^^,^^8^^,^^27^^,^^29^^,^^32^^–^^41^^,^^44^^–^^47^^,^^50^^,^^51^^,^^53^^,^^56^^,^^62^	2 327	378 994	51 (33–78)	99.4
≥ 10 000 pregnancies	12^7^^,^^8^^,^^27^^,^^29^^,^^32^^,^^39^^,^^41^^,^^44^^,^^46^^,^^47^^,^^50^^,^^56^	1 662	293 657	42 (21–81)	99.7

CI: confidence interval; GDP: gross domestic product; WHO: World Health Organization.

Although our leave-one-study-out analysis highlighted the stability of the pooled incidence estimate (85–96 cases per 10 000 pregnancies; *I*^2^: 98.6–99.2%; online repository),^20^ the exclusion of low-quality and then both low- and medium-quality studies increased the estimate to 94 (95% CI: 64–139; *I*^2^: 99.2%)^6^^,^^8^^,^^12^^–^^15^^,^^27^^,^^29^^–^^33^^,^^35^^–^^42^^,^^44^^–^^53^^,^^55^^,^^56^^,^^58^^–^^62^ and 144 (95% CI: 86–241; *I*^2^: 99.5%),^6^^,^^8^^,^^13^^,^^27^^,^^31^^,^^37^^,^^39^^,^^41^^,^^44^^–^^46^^,^^52^^,^^53^^,^^56^^,^^58^^,^^59^ respectively, suggesting higher estimates from higher-quality studies. We observed that the estimates also decreased substantially to 51 (95% CI: 33–78; *I*^2^: 99.4%)^7^^,^^8^^,^^27^^,^^29^^,^^32^^–^^41^^,^^44^^–^^47^^,^^50^^,^^51^^,^^53^^,^^56^^,^^62^ and 42 (95% CI: 21–81; *I*^2^: 99.7%)^7^^,^^8^^,^^27^^,^^29^^,^^32^^,^^39^^,^^41^^,^^44^^,^^46^^,^^47^^,^^50^^,^^56^ when only considering studies with at least 5000 or 10 000 pregnancies, respectively (Table 2).

We did observe trends in socioeconomic factors: incidence was higher in low-income countries (113; 95% CI: 48–264; *I*^2^: 98.6%)^12^^,^^50^^,^^51^^,^^53^^,^^55^^–^^57^^,^^59^^,^^62^ than in lower-middle- (84; 95% CI: 54–131; *I*^2^: 98.4%)^6^^,^^7^^,^^13^^–^^15^^,^^27^^,^^29^^–^^42^^,^^47^^–^^49^^,^^52^^,^^58^^,^^60^ and upper-middle-income countries (94; 95% CI: 32–278; *I*^2^: 99.8%),^8^^,^^44^^–^^46^^,^^61^ but these lacked statistical significance. Countries in which health-care expenditure was less than 5% of GDP had higher estimates (94; 95% CI: 60–147; *I*^2^: 99.2%)^6^^–^^8^^,^^12^^–^^15^^,^^27^^,^^29^^–^^42^^,^^44^^,^^49^^,^^51^^–^^53^^,^^56^^–^^58^^,^^62^ compared to countries spending 5–10% of GDP on health care (82; 95% CI: 44–152; *I*^2^: 98.7%).^45^^–^^48^^,^^50^^,^^55^^,^^59^^–^^61^ Finally, we observed that incidence was lower in countries in which 75% or more of pregnant women received at least one antenatal care visit (81; 95% CI: 55–119; *I*^2^: 99.2%)^6^^–^^8^^,^^14^^,^^15^^,^^27^^,^^29^^–^^42^^,^^44^^–^^50^^,^^53^^,^^57^^–^^60^ compared to countries in which less than 75% of pregnant women received at least one antenatal care visit (146; 95% CI: 54–390; *I*^2^: 99.1%; Table 2).^12^^,^^13^^,^^51^^,^^52^^,^^55^^,^^56^^,^^61^^,^^62^

### Maternal and fetal outcomes

**Fig. 3:**
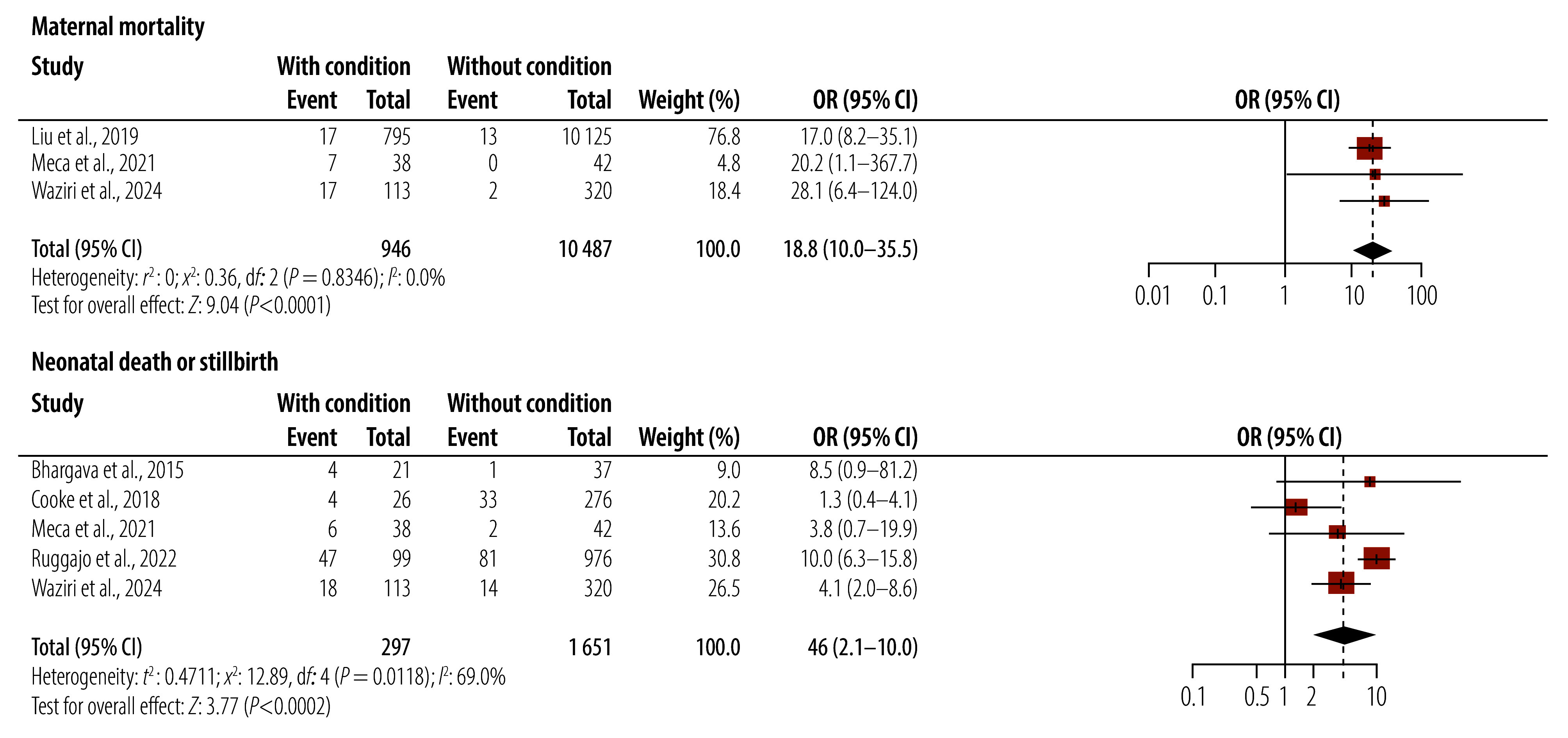
Individual study estimates and pooled effect of pregnancy-induced acute kidney injury on maternal mortality (upper) and neonatal death or stillbirth (lower) in low- and middle-income countries

CI: confidence interval; OR: odds ratio.

The incidence of pregnancy-associated acute kidney injury significantly affected maternal morbidity and both maternal and fetal outcomes. Dialysis was required in 29.1% of cases (95% CI: 18.6–42.6; *I*^2^: 92.9%),^7^^,^^8^^,^^13^^–^^15^^,^^27^^,^^30^^–^^42^^,^^44^^–^^53^^,^^55^^,^^56^^,^^59^^–^^62^ while 19.1% (95% CI: 11.8–29.4; *I*^2^: 88.4%)^6^^,^^13^^,^^15^^,^^30^^,^^32^^,^^34^^,^^36^^,^^38^^,^^39^^,^^41^^,^^44^^,^^47^^,^^48^^,^^51^^,^^53^^,^^55^^–^^57^^,^^59^^,^^60^^,^^62^ progressed to chronic or end-stage kidney disease. We estimated maternal mortality from the condition at 8 per 10 000 pregnancies (95% CI: 5–13; *I*^2^: 92.3%),^6^^–^^8^^,^^13^^–^^15^^,^^27^^,^^29^^–^^42^^,^^44^^,^^45^^,^^47^^–^^53^^,^^55^^–^^62^ accounting for 10.8% (95% CI: 7.6–15.3; *I*^2^: 85.1%) of all cases of pregnancy-associated acute kidney injury. Neonatal death or stillbirth occurred in 29.8% of all cases (95% CI: 24.2–36.1; *I*^2^: 81.6%; Table 3).^6^^,^^8^^,^^15^^,^^27^^,^^29^^–^^36^^,^^38^^–^^40^^,^^42^^,^^44^^–^^46^^,^^48^^,^^49^^,^^51^^–^^53^^,^^55^^–^^62^ Compared with pregnancies without the condition, having pregnancy-associated acute kidney injury was associated with a significant 18.8-fold higher odds of maternal death (95% CI: 10.0–35.5; *I*^2^: 0.0%)^8^^,^^52^^,^^61^ and a 4.6-fold higher odds of adverse fetal outcomes (95% CI: 2.1–10.0; *I*^2^: 69.0%; Fig. 3).^6^^,^^52^^,^^53^^,^^59^^,^^61^

**Table 3:** Etiology and maternal and fetal outcomes in pregnancy-associated acute kidney injury in low- and middle-income countries

Etiology or outcome	No. studies reporting an event	No. events within relevant studies		Pregnancy-associated acute kidney injury				Pregnancies		
				No. cases within relevant studies	Pooled prevalence (95% CI)	*I*^2^, %		No. pregnancies within relevant studies	Pooled prevalence per 10 000 pregnancies (95% CI)	*I*^2^, %
**Outcome**										
Requiring haemodialysis	35^7^^,^^8^^,^^13^^–^^15^^,^^27^^,^^30^^–^^42^^,^^44^^–^^53^^,^^55^^,^^56^^,^^59^^–^^62^	716		3001	29 (19–43)	92.9		402 411	18 (12–29)	96.6
Maternal mortality	38^6^^–^^8^^,^^13^^–^^15^^,^^27^^,^^29^^–^^42^^,^^44^^,^^45^^,^^47^^–^^53^^,^^55^^–^^62^	350		3006	11 (8–15)	85.1		351 994	8 (5–13)	92.3
Progression to chronic or end-stage kidney disease	21^6^^,^^13^^,^^15^^,^^30^^,^^32^^,^^34^^,^^36^^,^^38^^,^^39^^,^^41^^,^^44^^,^^47^^,^^48^^,^^51^^,^^53^^,^^55^^–^^57^^,^^59^^,^^60^^,^^62^	345		1335	19 (12–29)	88.4		160 710	18 (12–26)	92.8
Neonatal death or stillbirth	32^6^^,^^8^^,^^15^^,^^27^^,^^29^^–^^36^^,^^38^^–^^40^^,^^42^^,^^44^^–^^46^^,^^48^^,^^49^^,^^51^^–^^53^^,^^55^^–^^62^	785		2678	30 (24–36)	81.6		323 420	24 (16–36)	97.0
**Etiology**										
Sepsis	33^7^^,^^8^^,^^13^^–^^15^^,^^27^^,^^29^^–^^32^^,^^34^^–^^41^^,^^44^^–^^46^^,^^48^^–^^53^^,^^55^^,^^56^^,^^59^^–^^62^	537		2882	17 (11–23)	92.0		393 259	11 (7–18)	93.8
Pre-eclampsia	33^7^^,^^8^^,^^13^^–^^15^^,^^27^^,^^29^^–^^32^^,^^34^^–^^41^^,^^44^^–^^46^^,^^48^^–^^53^^,^^55^^,^^56^^,^^59^^–^^62^	1317		2882	44 (37–52)	89.8		393 259	31 (20–48)	97.9
Haemorrhage	32^7^^,^^13^^–^^15^^,^^27^^,^^29^^–^^32^^,^^34^^–^^41^^,^^44^^–^^46^^,^^48^^–^^53^^,^^55^^,^^56^^,^^59^^–^^62^	578		2162	26 (22–31)	81.2		382 339	18 (12–26)	95.9

CI: confidence interval.

We observed reduced heterogeneity on conducting a subgroup analysis by country and WHO region (lowest *I*^2^ = 0.0% and 66.8%, respectively), with the highest maternal and fetal mortality in the African Region of 13 (95% CI: 4–44) and 49 (95% CI: 24–100) deaths per 10 000 pregnancies, respectively. Other socioeconomic factors did not significantly explain heterogeneity (online repository).^20^

### Etiologies

Thirty-three studies^7^^,^^8^^,^^13^^–^^15^^,^^27^^,^^29^^–^^32^^,^^34^^–^^41^^,^^44^^–^^46^^,^^48^^–^^53^^,^^55^^,^^56^^,^^59^^–^^62^ reported the etiology or risk factors of pregnancy-associated acute kidney injury. However, only eight studies specified diagnostic criteria (online repository).^20^ The most common causes were pre-eclampsia (44.1%; 95% CI: 36.6–52.0; *I*^2^: 89.8%),^7^^,^^8^^,^^13^^–^^15^^,^^27^^,^^29^^–^^32^^,^^34^^–^^41^^,^^44^^–^^46^^,^^48^^–^^53^^,^^55^^,^^56^^,^^59^^–^^62^ haemorrhage (26.2%; 95% CI: 21.8–31.3; *I*^2^: 81.2%)^7^^,^^13^^–^^15^^,^^27^^,^^29^^–^^32^^,^^34^^–^^41^^,^^44^^–^^46^^,^^48^^–^^53^^,^^55^^,^^56^^,^^59^^–^^62^ and sepsis (16.5%; 95% CI: 11.4–23.4; *I*^2^: 92.0%),^7^^,^^8^^,^^13^^–^^15^^,^^27^^,^^29^^–^^32^^,^^34^^–^^41^^,^^44^^–^^46^^,^^48^^–^^53^^,^^55^^,^^56^^,^^59^^–^^62^ corresponding to 31 (95% CI: 20–48; *I*^2^: 97.9%), 18 (95% CI: 12–26; *I*^2^: 95.9%) and 11 (95% CI: 7–18; *I*^2^: 93.8%) cases per 10 000 pregnancies, respectively (Table 3). Heterogeneity was high (*I*^2^: 81.2–97.9%), prompting further subgroup analyses. Incidences varied by country and WHO region, with sepsis- and pre-eclampsia-related pregnancy-associated acute kidney injury highest in Nigeria and Chad, respectively (online repository).^20^

### Socioeconomic factors and trend interaction 

**Fig. 4:**
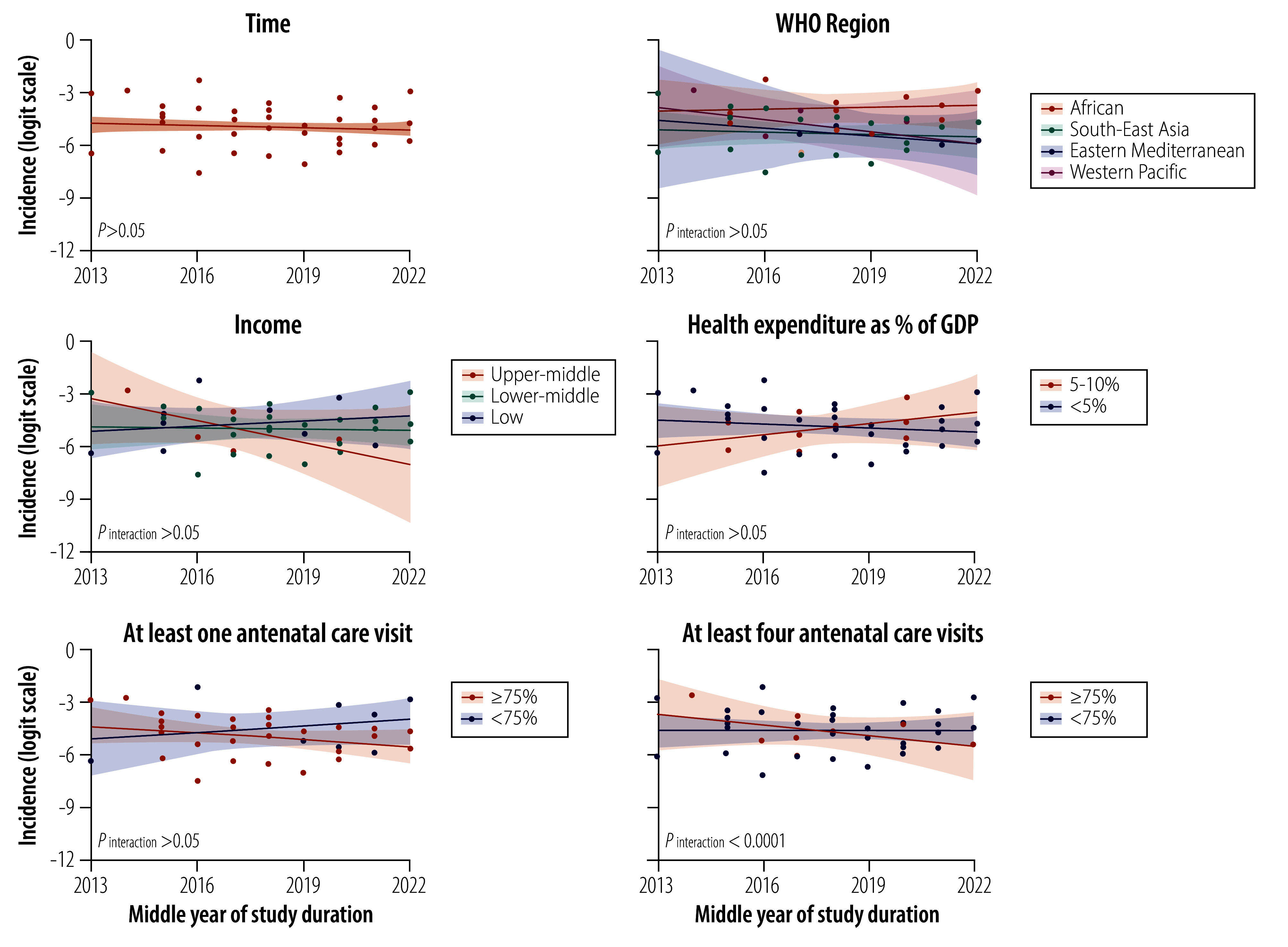
Interaction between socioeconomic factors and middle year of data acquisition as moderators of the incidence of pregnancy-associated acute kidney injury in low- and middle-income countries

Although we observed a slight decline in the overall trend of pregnancy-associated acute kidney injury from 2013 to 2023 (using the Wald z-test *P*-value > 0.05), multivariable meta-regression revealed different trends across subgroups. Among the WHO regions, only the studies conducted in the African Region showed a slight increase (studies conducted in the South-East Asia, Eastern Mediterranean and Western Pacific regions showed declines). By income group, studies conducted in upper-middle-income countries exhibited a steep decline, whereas those in low-income countries showed a slight increase. Studies based in countries in which health-care spending was 5–10% of GDP demonstrated an increasing trend, while those in which health-care spending was less than 5% of GDP showed a decline. However, none of these interactions was statistically significant, with *P*-values of more than 0.05 (Fig. 4).

GDP: gross domestic product.

Notably, the trend in the incidence of pregnancy-associated acute kidney injury varied significantly by the availability of antenatal care. Countries in which 75% or more of pregnant women received at least four antenatal care visits showed a declining trend, while those in which less than 75% of pregnant women received at least four antenatal care visits exhibited an increasing trend (*P*_interaction_ < 0.0001; Fig. 4). This factor also had a significant moderating effect on related maternal and fetal mortality (*P*_interaction_ < 0.0001; online repository),^20^ with a slower increase in maternal mortality and a sharper decline in neonatal death or stillbirth observed in countries in which 75% or more of pregnant women received at least four antenatal care visits. These findings remained robust in sensitivity analyses, including modelling as a continuous variable and adjusting for study design and quality (online repository).^20^

## Discussion

Our meta-analysis of pregnancy-associated acute kidney injury in low- and middle-income countries revealed a substantial burden. One quarter of women with the condition required dialysis, and one fifth progressed to chronic or end-stage kidney disease. One tenth of cases resulted in maternal death, and around one third in neonatal death or stillbirth. 

By extrapolating our findings to approximately 190 million pregnancies annually worldwide,^63^ pregnancy-associated acute kidney injury may affect 1.7 million pregnancies and lead to more than 186 000 deaths in low- and middle-income countries. Our findings align with previous work that focused on specific regions. Case fatality rates reached 34.4% in Africa, with most women requiring intensive care and haemodialysis.^64^ In Asia, case fatality rates varied between 7.7% (10/130) and 28.9% (24/83), and up to 50% (16/32) of women had an outcome of neonatal death or stillbirth.^1^ A meta-analysis comparing nearly 8000 pregnancies with and without the condition found that acute kidney injury was associated with a more than fourfold increase in the risk of maternal death, a longer stay in intensive care by 2 days, a threefold increased incidence of stillbirth or perinatal death, and a birth weight lower by 740 g.^2^ These results are consistent with WHO data showing a global maternal mortality of 223 deaths per 100 000 live births in 2020, with nearly 95% of maternal deaths occurring in low-resource settings.^65^

Although our subgroup analysis did not show a noticeable difference in the incidence of pregnancy-associated acute kidney injury between low- and upper-middle-income countries, the gap is large when comparing with data from high-income countries. At 12 reported cases per 10 000 pregnancy-related hospitalizations in the USA,^5^ our pooled findings indicate an incidence of eight times higher for low- and middle-income countries and 20 times higher for countries within the WHO African Region. Similarly, our pooled proportion of cases receiving dialysis and maternal mortality are threefold higher than those reported in the USA (8.2%; 351/4300 and 4.0%; 170/4300, respectively).^5^

Systemic health-care disparities, inadequate access to prenatal care, delayed interventions, limited availability of kidney replacement therapy and other resource constraints are factors that likely drive the disproportionate burden in low- and middle-income countries. Low preparedness for acute kidney injury care, as highlighted in a recent report by the International Society of Nephrology, could also be a contributing factor. The report indicated that registries and advocacy groups are mostly unavailable, and nephrologists are alarmingly low in low-income countries.^66^

We identified pre-eclampsia, haemorrhage and sepsis as leading etiologies, as well as an association between greater antenatal care coverage and decreasing incidence, consistent with the WHO publication on leading maternal mortality causes^67^ as well as systematic reviews conducted in African and Asian countries.^64^^,^^68^ Fragmented prenatal care, delayed diagnosis and inadequate referral systems exacerbate these conditions, perhaps allowing them to progress to acute kidney injury. The burden of infectious diseases also remains high in low- and middle-income countries, with conditions such as human immunodeficiency virus infection, malaria, leptospirosis and septic abortion being important contributors to pregnancy-associated acute kidney injury. Unsafe abortion practices, often resulting from restrictive reproductive health laws and limited access to contraception, are still prevalent in several low- and middle-income countries, and continue to lead to sepsis-related acute kidney injury during pregnancy.^31^^,^^45^ These challenges are rarely encountered in high-income countries, where reproductive health services are generally safer and more accessible. Nevertheless, the fact that these conditions are all deemed preventable or treatable offers important opportunities for improvement.

Our findings that higher antenatal care availability was significantly associated with lower incidence and improved maternal and fetal outcomes emphasize the importance of adequate antenatal care. Frequent antenatal care improves pregnancy-associated acute kidney injury outcomes through early detection, risk mitigation and timely intervention, and enables early management of hypertensive disorders such as pre-eclampsia via blood pressure monitoring, antihypertensives and preventive eclampsia therapy.^69^ Prompt diagnosis of infections such as sepsis allows timely antibiotic treatment, reducing inflammation and kidney damage.^70^ Antenatal care also mitigates haemorrhage risks by screening for anaemia, providing iron supplementation and implementing postpartum haemorrhage protocols to prevent hypovolemia-induced acute kidney injury.^71^

Structured antenatal care also improves fetal outcomes by reducing preterm births and low birth weight, both linked to complications of pregnancy-associated acute kidney injury. Studies show regular antenatal care attendees have lower risk of perinatal death as a result of better maternal health.^52^ Antenatal care also facilitates timely referrals to tertiary care, critical for accessing renal replacement therapy (although low- and middle-income countries often face dialysis shortages).^72^ By integrating risk stratification and patient education, antenatal care empowers women to seek care earlier, mitigating delays that exacerbate the severity of pregnancy-associated acute kidney injury in low-resource settings.^73^

Our study has several strengths. Our comprehensive literature review included all the main databases as well as several sources of grey literature. We minimized between-study variability and ensured generalizability by including only studies that used criteria defined by the Kidney Disease: Improving Global Outcomes organization. We explored the impact of geographic, socioeconomic and antenatal care factors on heterogeneity with our extensive subgroup analyses and meta-regression.

However, our study was limited by several factors. First, a potential source of heterogeneity among our reviewed studies is a variation in the definition of the pregnancy and postpartum period; for example, some studies included participants from 20 weeks of gestation to 6 weeks postpartum,^12^^,^^59^ although other studies only included participants up to the end of the immediate postpartum (i.e. 24 hours after giving birth)^57^ or for 2 weeks postpartum.^48^ Second, variability in denominators (e.g. total pregnancies, deliveries or live births) may have contributed to discrepancies. Third, although we attempted to include studies with harmonized condition definitions, Kidney Disease: Improving Global Outcomes criteria are not standard because serum creatinine is also known to be lowered during pregnancy; the true incidence of pregnancy-associated acute kidney injury could therefore be higher than estimated here.^43^ Fourth, studies conducted retrospectively versus prospectively may have been subject to recall bias, although we attempted to address this by subgroup analysis and multivariable meta-regression adjustment. Fifth, only a subset of the studies defined the etiology of the condition, and these definitions were inconsistent. Finally, we were not able to assess high-risk subgroups (e.g. those with prior pregnancy-associated acute kidney injury, autoimmune conditions or kidney disease) because of limited access to original data.

We have demonstrated that pregnancy-associated acute kidney injury is a significant maternal health concern in low- and middle-income countries. By providing more resources to prevent the common etiologies and expand the availability of antenatal care, the burden of this condition and its effects on maternal and fetal outcomes can be reduced. We anticipate that our findings will provide critical insights to inform targeted public health strategies, ultimately reducing preventable deaths in low-resource settings.
